# Supplementary material for: Complexome profiling of the Chlamydomonas psb28 mutant reveals TEF5 as an early PSII assembly factor
Source: Plant Cell. 2025 Mar 17;37(6):koaf055. doi: 10.1093/plcell/koaf055 (PMC12164590; doi:10.1093/plcell/koaf055)
Supplement: koaf055_Supplementary_Data [file koaf055_supplementary_data.zip › psb28_interactive.zip!/plots/intensity/qualityPlots/peptides/NUOS4_Cre03.g146247.t1.1.html]

### Cre03.g146247.t1.1

Trivial name: NUOS4  
SeqCoverage [median]: 71.30  
Mass: 17.39 kDa  
  
 

| Sequence | MissedCleavage | Mass [Da] | wtIntensity | muIntensity | Score |
| --- | --- | --- | --- | --- | --- |
| AAEITGAAESAMGPK | 0 | 1402.68 | 19191.43 | 14510.82 | 188.27 |
| AAEITGAAESAMGPKEGGFTAGVPLDTFTR | 1 | 2951.43 | 0.00 | 3054.46 | 59.28 |
| AGVPDLSTLPSNR | 0 | 1325.69 | 14420.92 | 44278.78 | 164.65 |
| EGGFTAGVPLDTFTR | 0 | 1566.77 | 26787.70 | 11871.69 | 164.48 |
| EGGFTAGVPLDTFTRK | 1 | 1694.86 | 637.65 | 2153.69 | 38.03 |
| IEFEPTAK | 0 | 933.48 | 13404.50 | 12763.58 | 127.84 |
| KAAEITGAAESAMGPK | 1 | 1530.77 | 3927.31 | 1937.27 | 93.18 |
| LGWEYEVTEPNK | 0 | 1463.69 | 965.21 | 0.00 | 75.29 |
| LGWEYEVTEPNKR | 1 | 1619.79 | 868.81 | 0.00 | 53.97 |
| RAGVPDLSTLPSNR | 1 | 1481.80 | 6145.65 | 7045.74 | 94.73 |
| SALVFYTK | 0 | 927.51 | 7615.29 | 6254.80 | 103.74 |
| SALVFYTKEEAMR | 1 | 1543.77 | 531.02 | 0.00 | 56.95 |
| TASQSGLAR | 0 | 889.46 | 26882.13 | 26476.71 | 174.92 |
| TVDFATTTPAWK | 0 | 1336.67 | 3013.26 | 0.00 | 94.31 |
| YMQYGDNFGTK | 0 | 1322.56 | 1608.33 | 1794.33 | 102.06 |
| YMQYGDNFGTKR | 1 | 1478.66 | 12456.63 | 12426.34 | 73.63 |
|  |  |  | 138455.8 | 144568.2 |  |
